# Intermediate-Length GGC Repeat Expansion in NOTCH2NLC Was Identified in Chinese Patients with Amyotrophic Lateral Sclerosis

**DOI:** 10.3390/brainsci13010085

**Published:** 2023-01-01

**Authors:** Mengxia Wan, Ji He, Junyan Huo, Can Sun, Yu Fu, Dongsheng Fan

**Affiliations:** 1 Department of Neurology, Peking University Third Hospital, Beijing 100191, China; 2 Beijing Municipal Key Laboratory of Biomarker and Translational Research in Neurodegenerative Diseases, Beijing 100191, China; 3 Key Laboratory for Neuroscience, Ministry of Education/National Health Commission, Peking University, Beijing 100191, China

**Keywords:** amyotrophic lateral sclerosis, *NOTCH2NLC*, intermediate-length repeats, nucleotide repeat expansion

## Abstract

GGC repeat expansions in the 5’ untranslated region (5’UTR) of the Notch Homolog 2 N-terminal-like C gene (*NOTCH2NLC*) have been reported to be the genetic cause of neuronal intranuclear inclusion disease (NIID). However, whether they exist in other neurodegenerative disorders remains unclear. To determine whether there is a medium-length amplification of *NOTCH2NLC* in patients with amyotrophic lateral sclerosis (ALS), we screened 476 ALS patients and 210 healthy controls for the presence of a GGC repeat expansion in *NOTCH2NLC* by using repeat-primed polymerase chain reaction (RP-PCR) and fragment analysis. The repeat number in ALS patients was 16.11 ± 5.7 (range 7–46), whereas the repeat number in control subjects was 16.19 ± 3.79 (range 10–29). An intermediate-length GGC repeat expansion was observed in two ALS patients (numbers of repeats: 45, 46; normal repeat number ≤ 40) but not in the control group. The results suggested that the intermediate *NOTCH2NLC* GGC repeat expansion was associated with Chinese ALS patients, and further functional studies for intermediate-length variation are required to identify the mechanism.

## 1. Introduction

Recently, increasing numbers of repeat-expansion-related genes were identified as the causes of neurological disease [1]. Among them, a trinucleotide repeat (GGC) abnormal expansion in the 5′-untranslated region of the Notch Homolog 2 N-Terminal-Like C gene (*NOTCH2NLC*) in chromosome 1 attracted substantial attention and was reported as the cause of neuronal intranuclear inclusion disease (NIID) in Chinese and Japanese studies [2,3,4]. The *NOTCH2NLC* gene is one of the three paralogs of the NOTCH2 receptor, which plays a vital role in human cortex expansion [5]. NIID is a rare disease mainly involving nervous symptoms and is pathologically characterized by the presence of eosinophilic, p62- and ubiquitin-positive intranuclear inclusions in cells of the skin, peripheral and central nervous systems, and other visceral organs [6]. Furthermore, pathogenic expansion can be detected in a small proportion of patients with other neurological diseases such as dementia, Parkinson’s syndrome, peripheral neuropathy, myopathy, leukoencephalopathy, and essential tremor.

In addition to finding new repeat-expansion-related genes and new links to various diseases, researchers have shown interest in the association between the length of repeat expansion and clinical types. Short repeats in *NOTCH2NLC* are associated with NIID Parkinson’s disease dominant subtype (NIID-P), while the intermediate and long expansion is linked to NIID dementia dominant subtype (NIID-D) and myasthenic dominant subtype (NIID-M), respectively [7]. Intermediate-length repeat expansion refers to repeat sizes between normal and pathogenic ranges and has not previously received enough attention. Generally, the normal repeat size is less than 40 times in the general population, and the abnormal repeat size is more than 40 times. The pathogenic repeat expansion mutation is a repeat size exceeding 60 repeats, and the intermediate length is a repeat size between 40 and 60 repeats [7].

Amyotrophic lateral sclerosis (ALS) is a type of neurodegenerative disease that presents with insidious muscle weakness and atrophy caused by upper and lower motor neuron degeneration in the brain and spinal cord [8]. Motor neuron degeneration causes severe limb weakness, muscle fasciculations, disturbed speech, dysphagia, and death due to respiratory failure 3–5 years after disease onset. The etiology and risk factors for ALS remain unclear. Various genetic and environmental factors have been identified to contribute to the development of ALS [9]. To date, more than 40 genes have been identified to be associated with amyotrophic lateral sclerosis, and *SOD1, TARDBP, FUS,* and *C9ORF72* are the most common pathogenic genes in familial ALS (FALS) [10]. The intermediate-length (G4C2)n repeat expansion in *C9ORF72* and the CAG repeat expansion in *ATXN2* are associated with ALS and increase sporadic ALS(SALS) risk [11,12]. Identifying ALS-related genes can help to understand the disease’s pathogenesis.

There are many similarities in clinical manifestations and pathological phenotypes between the NIID-M and ALS. For example, muscle weakness in limbs and ubiquitin- and p62-positive intranuclear inclusions can be found in NIID-M and ALS [6,13,14]. Furthermore, repeat-expansion-related genes play a vital role in ALS. Therefore, the association between the intermediate-length expansion of GGC in *NOTHCH2NLC* and ALS draws wide attention. In a study with 545 patients in mainland China, intermediate-length mutations were found [15]. However, in a study in Taiwan with 304 patients, no abnormal GGC expansion was found in ALS patients [16]. Owing to the clinical significance and current controversial results, further investigation is required to determine whether intermediate-length expansions specifically affect ALS.

In this study, we detected GGC repeat expansion in the *NOTCH2NLC* gene in a large cohort of Chinese ALS patients and investigated the relationship between *NOTCH2NLC* GGC repeat expansion and ALS. We found intermediate-length GGC repeat expansions in two ALS patients. The results suggested that the intermediate-length GGC repeat expansion in *NOTCH2NLC* was associated with ALS.

## 2. Methods

### 2.1. Subjects

The study included 476 Han Chinese ALS patients and 210 healthy controls from the Department of Neurology of Peking University Third Hospital between 2017 and 2020. All ALS cases met diagnostic criteria for ALS (the Escorial criteria [8]), with a diagnostic grade consistent with probable, suspected, or laboratory support-suspected diagnoses, and FALS patients with a family history were excluded. The patients had a negative gene screening test for *SOD1*, *TARDBP*, *FUS*, and *C9ORF72*. The control cohort was defined as people without possible neurodegenerative diseases, such as motor neuron disease, Alzheimer’s disease, and Parkinson’s disease, and a family history of the above diseases was excluded based on case records. The healthy control subjects were enlisted in the same area between 2017 and 2020. The age, sex, and race of the ALS group and the healthy control group were matched. The study was approved by the ethics committee of Peking University Third Hospital, and all participants signed informed consent forms. Study protocols were approved by the Ethics Committee review boards of Peking University Third Hospital. All human research in this study was conducted according to the Declaration of Helsinki.

### 2.2. Demographic and Clinical Data

Clinical data, including age, sex, history, family history, age of onset, and site of onset, were collected. Neurological examinations were administered by two neurologists experienced in treating ALS patients. The disease onset site was defined as bulbar onset or spinal onset. The Amyotrophic Lateral Sclerosis Functional Rating Scale (ALSFRS-R) was assessed to evaluate disease severity functional limitations in ALS patients. The Mini-Mental State Examination (MMSE) score, Edinburgh Cognitive and Behavior ALS Screen (ECAS), electromyogram (ECG), and cerebral fluid examinations were administered to some ALS patients. Demographic data in healthy controls, such as sex, age, and place of origin, were collected.

### 2.3. Blood Collection and DNA Extraction

Whole peripheral blood from participants was collected in ethylenediaminetetraacetic acid (EDTA) tubes. Genomic DNA was extracted using whole blood genomic DNA extraction kits (Aidlab Biotechnologies Co., Ltd, Beijing, China)DNA concentration and purity were assessed spectrophotometrically at 260 and 280 nm using a Nanodrop 2000. DNA was diluted to 20-100 ng/μL and used as a working solution.

### 2.4. Polymerase Chain Reaction Analysis

The *NOTCH2NLC* GGC repeat size was analyzed as previously described [3] but with small modifications. We used fragment analysis to identify the size of the fragment and repeat-primed PCR (RP-PCR) to identify whether it had abnormal expansions. For the fragment analysis, the PCR primer mix contained two primers, 0.3 μM *NOTCH2NLC*-F( 5′-FAM- CATTTGCGCCTGTGCTTCGGAC-3′) and 0.3 μM *NOTCH2NLC*-R (5′-AGAGCGGCGCAGGGCGGGCATCTT-3′). For RP-PCR, the PCR primer mix contained three primers: 0.3 μM *NOTCH2NLC*-F(5′-FAM- GGCA TTTG CGCC TGTG CTTCGGACCGT-3′), 0.3 μM M13-(GGC)4(GGA)2 R (5′- CAGGAAAC AGCT ATGA CCTC CTCC GCCG CCGCCGCC-3′, and 0.3 μM M13-linker-R(5′-CAGGAAACAGCTATGACC-3′). The PCR mix contained 0.25 U PrimeSTAR GXL DNA Polymerase, 1× PrimeSTAR GXL Buffer, 200 μM each dNTP Mixture (Takara Biomedical Technology (Beijing) Co., Ltd., Beijing, China), 5% dimethyl sulfoxide (sigma Aldrich (Shanghai) Trading Co., Ltd. Shanghai, China), 1 M betaine (Sigma-Aldrich), 0.3 μM each primer mix and 20–100 ng genomic DNA in a total reaction volume of 10 μL. For the two-primer PCR, the initial denaturation temperature was set at 98 °C for 10 min, then 30 cycles were initiated: 8 °C for 30 s, 58 °C for 1 min, and 68 °C for 2 min, followed by a final elongation step of 68 °C for 10 min. For the RP_PCR, the cycling conditions were set as follows: 16 cycles of 98 °C for 30 s, 72 °C for 15 s with a reduction of 0.5 °C per cycle, and 68 °C for 30 s, and 29 cycles of 98 °C for 30 s, 62 °C for 15 s and 68 °C for 30 s, followed by a final elongation step of 68 °C for 10 min. All PCR products were collected for capillary electrophoresis.

### 2.5. Capillary Electrophoresis

Electrophoresis was performed on a 3730xl DNA Analyzer (Applied Biosystems Inc, Foster City, USA) using the 500 LIZ dye Size Standard, and the data were analyzed by GeneMarker software. For the fragment analysis, the length of the highest signal peak of two-primer PCR product capillary electrophoresis in two alleles was used to calculate the repeat GGC size, and the larger one was used as the repeat size of the participants, as NIID is dominantly inherited. The calculation method was applied according to the results of first-generation sequencing in a standard patient and reference human genome hg38. Capillary electrophoresis of the RP-PCR product was used to identify the result, and a sawtooth tail pattern in the electropherogram was judged as abnormal repeat expansion.

### 2.6. Statistical Analysis

SPSS 22 was used for data processing. For measurement data, those with a normal distribution after inspection were expressed as the mean ± standard deviation (x ± s). To explore the correlation between CSF protein level and GGC repeat size, Spearman’s test was used. A two-tailed *p* < 0.05 was considered to demonstrate statistical significance.

## 3. Results

### 3.1. Clinical Data

A total of 476 patients were involved in this cohort, including 324 males and 152 females, and the control participants consisted of 120 males and 90 females. The mean age of ALS patients was 53.21 ± 11.66, while the age at enrollment for the control cohort was 52.58 ± 11.73. In total, 389 patients (81.7%) had initial spinal cord involvement, while 87 ALS patients (18.2%) had bulbar involvement initially. No patient had initial respiratory muscle involvement. A summary of the clinical features and demographic information of ALS and control participants is presented in Table 1.

### 3.2. (GGC)n Expansion of NOTCH2NLC

**Figure 1:**
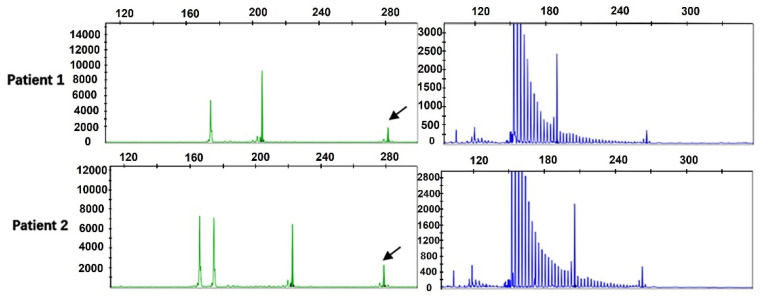
Fragment analysis (green) and repeat-primed PCR (blue) for two ALS patients harboring GGC intermediate-length repeat expansions. Fragment analysis showed the fragment size and the length of the highest signal peak (arrows). Repeat-primed PCR showed a sawtooth tail pattern of repeat expansion.

By performing capillary electrophoresis of the two-primer PCR and repeated primer PCR product, we found that the repeat size was between 7 and 46 in the 476 ALS patients, and the mean number of repeats was 16.11 ± 5.7. Among them, 2 ALS cases (4.20%) had more than 40 repeats, and the repeats were 45 times and 46 times. RP-PCR revealed the sawtooth pattern, which identified abnormal repeat expansion (Figure 1).

**Figure 2:**
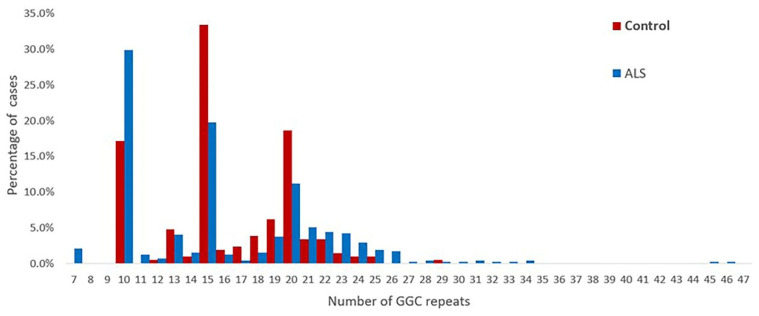
Distribution of the sizes of the NOTCH2NLC GGC repeats in 476 patients with ALS and 210 healthy controls.

The repeat numbers in the controls were all less than 40 times, the repeat number was 10–29 in the controls, and the mean number of repeats was 16.19 ± 3.79. The distribution of the repeat sizes for the ALS patients and controls is shown in Figure 2.

### 3.3. Clinical Features

We summarized the clinical data of two identified ALS patients with intermediate-length GGC repeat, and the details are listed in Table 2.

Patient 1 was a 52-year-old male who carried 46 repeats. He initially presented with muscle weakness in the right foot at 50 years of age. Then, he showed muscle weakness in the four limbs and dysarthria gradually in the following two years. Neurological examination showed increased tendon reflexes in all extremities, and the Babinski sign, Hoffmann sign, abdominal reflex, and Maxillofacial reflex were all positive. Altogether, damage existed in the four upper motor units of the bulbar, cervical, thoracic and lumbar segments and three lower motor units of the bulbar, cervical, and lumbar segments. The ECAS score was 83, which is in the normal range. Brain magnetic resonance imaging (MRI) showed unspecific periventricular and subcortical white matter lesions (image unavailable). At that time, electromyography showed abundant and diffuse ongoing denervation (spontaneous potentials) and chronic reinnervation changes in the cervical, thoracic and lumbar segments. Sensory conduction studies showed compound muscle action potential (CMAP) reduction in both the ulnar and right median nerves. Pulmonary function indicated restrictive ventilatory dysfunction, the residual capacity ratio increased, and forced vital capacity (FVC) was 52%. Cerebrospinal fluid (CSF) examination showed elevated protein 338 mg/L, and oligoclonal bands were suspiciously positive both in the CSF and blood. He was clinically diagnosed with definite ALS in the third stage of KCSS and then died of respiratory failure 35 months after onset. Systemic examinations on tumor and immunological disease, including immunological index, tumor marker, and positron emission tomography computer tomography (PET-CT), were performed to exclude ALS mimics. Besides Riluzole, he had been treated with Intravenous Immunoglobulin Gamma (IVIG) because of his insistence. Nevertheless, IVIG did not improve his condition and prognosis.

**Figure 3:**
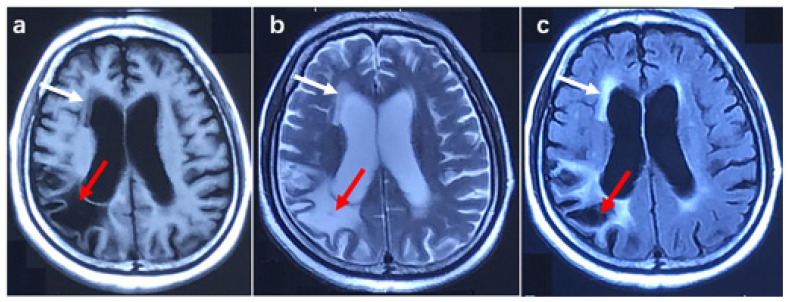
Brain MRI image in patient 2. (**a**) T1 weighted image, (**b**) T2 weighted image, (**c**) T2 Fluid-attenuated inversion recovery image. The red arrow shows malacia in the right parietal lobe, and the white arrow indicates a white matter lesion.

Patient 2 was a female with 45 GGC. She complained of muscle weakness in both upper limbs at the age of 62. In the following two years, she suffered from muscle weakness in both lower limbs, dysarthria, and dysphagia. The patient was psychologically unstable in the past, regularly taking risperidone. In addition, she had transient cognitive impairment and urinary incontinence at the age of 61. Six months after onset, the physical examination showed damage in the three upper motor units of the bulbar, thoracic, and lumbar segments and one lower motor unit of the cervical segments. The cognitive function examination showed an ECAS score of 49 points and an MMSE score of 22 points, indicating cognitive impairment. Electromyography showed decreased amplitude in both upper extremity nerve motor conduction and the spontaneous potential of the bulbar and cervical segments. Brain MRI showed mild white matter lesions and lesions in the right parietal lobe, brain atrophy, and an enlarged ventricle (shown in Figure 3). According to history and imaging features, the lesion in the right parietal lobe was malacia due to cerebral infarction in the past.

Thorough blood and CSF examinations were completed to exclude ALS mimics and other similar diseases. Unfortunately, diffusion-weighted image sequencing, which helps to identify NIID by exhibiting hyperintensities at the corticomedullary junction, was not performed as a result of the under-recognition of NIID at that time. CSF examination showed elevated protein 565.0 mg/L↑, and oligoclonal bands were suspiciously positive both in the CSF and blood, similar to patient 1. Memantine was administered to improve the patient’s cognitive function. She was conclusively clinically diagnosed with ALS and then died of respiratory failure at 64 years of age, 30 months after the disease onset.

In summary, clinically, two patients presented with limbal muscle weakness and gradually developed more severe limb weakness, dysarthria, and dyspnea. Examination showed upper motor neuron signs in two patients (4 and 3 segments). Additionally, patient 2 presented with cognitive dysfunction, and two patients had mild white matter lesions, although we do not know if they were related to the intermediate-length expansion. No obvious abnormalities were identified in blood immunological function, inflammatory index, or serum tumor markers. No unusual findings were detected by electrocardiography or chest CT. Elevated protein was identified in the CSF, and suspicious oligoclonal bands were detected in the CSF and blood.

## 4. Discussion

In this study, we aimed to study the role of intermediate-length GGC repeat expansion in *NOTCH2NLC* in Chinese ALS patients. Therefore, we screened the *NOTCH2NLC* gene in 476 ALS patients and 210 controls and identified 2 intermediate-length GGC repeats 45 and 46 times in ALS patients and none in the control subjects, indicating that intermediate-length GGC repeat expansion is associated with ALS.

It has been shown that the GGC repeat expansion in *NOTCH2NLC* is associated with many neurologic disorders, such as Parkinson’s disease, frontal temporal dementia, and essential tremor [17]. The GGC repeat size varies in NIID phenotypes and different neurological diseases [7]. The normal GGC range in *NOTCH2NLC* was 5–38 in healthy controls, while a repeat size > 60 indicated pathogenic GGC expansion [2]. More than 200 repetitions may increase susceptibility to NIID-M, and fewer than 100 repetitions may be associated with Parkinson’s disease [7]. A total of 41-60 GGC repeats in *NOTCH2NLC* were defined as intermediate-length repeat expansions [18], which have been identified in many neurodegenerative diseases, such as Parkinson’s disease, leukoencephalopathy, and Alzheimer’s disease [2,18,19,20,21,22]. For Parkinson’s disease, they found 11 patients with 41–52 repeats and 7 patients with 41–64 repeats in China and Singapore, respectively [18,19]. Fibroblasts from PD patients harboring intermediate-length repeat expansions revealed *NOTCH2NLC* upregulation and autophagic dysfunction, which suggested that medium-length repeat expansions in *NOTCH2NLC* were associated with PD [18]. Intermediate-length GGC repeats 42–58 and 42–47 times were found in patients with Alzheimer’s disease and leukoencephalopathy, respectively [21,22].

For ALS, Yuan et al. [15] found that the repeat size ranged from 7 to 143 units and that GGC repeat expansion accounted for approximately 0.73% (4/545) of all ALS patients. They concluded that ALS was a specific phenotype of NIID or that GGC expansion in *NOTCH2NLC* was a modifiable factor for ALS. Similar to this study, intermediate GGC repeat expansion was also found in 2 ALS patients carrying 44 and 54 repeats. Nevertheless, no GGC repeat expansion was found in another study in Taiwan [16]. Our study provides evidence of the association between GGC repeat expansion and ALS. There are also other repeat-expansion-related genes linked to ALS risk. Ataxin-2 intermediate-length polyglutamine expansion is associated with increased ALS risk [12], and C9ORF72 intermediate-length repeat expansion is associated with corticobasal degeneration and ALS [11,23]. The significance of intermediate duplications, therefore, needs to be emphasized, and further studies in larger populations are needed to determine appropriate thresholds for pathogenic and normal duplications.

There are similarities in ALS patients with abnormal repeat expansion. There were more than three segments of the upper motor unit and lower motor unit dysfunction in these two patients with intermediate repeat expansion. The two patients both met the diagnostic criteria determined by the EI Escorial and Gold coast, and other diseases mimicking ALS were excluded [24]. We noted cognitive impairment in Patient 2 with 45 GGC repeats, similar to the two patients with 96 and 143 GGC repeat expansions in a previous study [15], who presented with memory impairment and behavioral impairment. In addition, a brain MRI showed mild white matter lesions in two patients, although the change was not specific. Mild leukoencephalopathy was previously found in patients with 54 GGC repeat expansions [15]. NIID patients mainly manifest cognitive dysfunction and leukoencephalopathy [6]. Another expansion disorder-related gene, C9ORF72, is the most common cause of familial frontotemporal dementia and ALS [25]. Although the current evidence is insufficient, these findings suggest that abnormal intermediate-length GGC repeats may be associated with cognitive impairment and white matter lesions in ALS.

The elevated CSF protein also attracted our attention. However, the literature shows that elevated CSF protein can be detected in both ALS and NIID [6]. Furthermore, we explored the correlation between CSF protein level and repeat number using the available CSF protein data in this ALS cohort. The results showed that the CSF protein level was not related to GGC repeat size using the Spearman correlation test. A previous study found that the CSF protein level determines a poor prognosis for spinal amyotrophic lateral sclerosis [26]. The cause and role of elevated CSF protein levels in ALS remain unclear, and the relationship between elevated CSF protein levels and intermediate-length expansion has not been studied. We can pay more attention to ALS patients with elevated CSF protein levels.

The overlap between NIID and ALS increased the difficulty of making a correct diagnosis. *NOTCH2NLC* repeat expansion was not found in the previous Taiwanese ALS cohort [16]. In addition, there are case reports that NIID may mimic the manifestations of ALS [27], and GGC repeat expansions in *NOTCH2NLC* (repeat size 248) lead to lower motor neuron syndrome [28]. Therefore, we need to be vigilant about whether the clinical diagnosis of ALS patients with abnormal or intermediate-length GGC expansion found by us was truly ALS. We can distinguish NIID-M from ALS by the following aspects, as Yuan et al. previously described [15]. First, ALS is often accompanied by damage in upper motor neurons, which leads to increased tendon reflexes and positive pathological signs, such as the Babinski sign and Hoffmann sign. Second, the prognosis of NIID-M is relatively better than that of ALS, and the disease duration is 16.6 and 3–5 years, respectively. Third, electromyography and nerve conduction studies provide useful information to make the correct diagnosis. The fibrillations and positive sharp-wave potentials cannot always exist in NIID-M patients, especially in the early stages of the disease. However, there are abundant and diffuse spontaneous potentials in ALS due to denervation. The performance in the nerve conduction study also differs in the NIID-M and ALS patients. Slow motor nerve conduction velocity (MCV) and sensory nerve conduction velocity (SCV) are more common in NIID-M. When considering the diagnosis of our two patients in this study, *NOTCH2NLC* can help us. The GGC repeat size in NIID is more than 60 times, and the repeat size in muscle weakness-dominant NIID is generally more than 200 times [2,3,4], while the GGC repeat size in the patients we reported is relatively small and cannot meet the repeat expansion criteria in NIID. In addition, the NIID case that mimics ALS has prominent lower motor neuron impairment, and upper motor unit impairment is not obvious [27], different from our patients, who have a three-regional upper and lower motor unit impairment. Therefore, enough evidence supports the ALS diagnosis.

The pathogenesis of GGC expansion in the *NOTCH2NLC* gene is currently unclear, and researchers have summarized the possible mechanisms, including (1) the toxicity of polyglycine-containing protein; (2) the toxicity of repeat RNA; (3) the GGC repeat size of *NOTCH2NLC*; (4) the size and types of trinucleotide interruption; and (5) the methylation status of *NOTCH2NLC* [7]. The current evidence mainly supports the formation of polyglycine-containing proteins. Recently, it has been certified that GGC trinucleotide repeats are translated into a polyglycine protein (N2NLCpolyG) through the upstream reading frame (uORF). N2NLCpolyG is rapidly degraded under normal conditions without GGC repeat expansion and does not produce aggregation, while the repeat expansion of GGC carried by the patient increases glycine numbers, which significantly enhances the stability and spontaneous aggregation of N2NLCpolyG, resulting in the formation of abnormal inclusion body-like protein aggregation [29,30]. Furthermore, N2NLCpolyG inclusions formed when GGC repeats expanded beyond 30, which may be one reason for the pathogenicity of medium-length expansions.

There were some limitations in our study. First, some important examinations were unavailable as the two patients passed away. Significant intensity signals in the DWI sequence in the corticomedullary junction and the inclusion body in the nervous system and skin were powerful cues for NIID diagnosis [6]. Nevertheless, we cannot obtain the two above examination results to provide more evidence to further distinguish ALS and NIID. Furthermore, the examination also helps to investigate the pathologic and imaging features in ALS with intermediate-length GGC expansions. Additionally, the number of control cases was 210 less than the ALS cohort, as the normal repeat size had been identified in other studies. Last, we did not perform experimental research to explore the mechanism of action of intermediate-length GGC repeats in *NOTCH2NLC* in ALS, and only two patients with intermediate-length GGC expansion were found, although the number of the cohort is not small regarding the incidence of ALS. In addition, it has also been reported that patients carrying intermediate repeat numbers are found in ALS patients [15], and in combination with our literature and reports from others, we believe that our speculation is justified. To identify the clinical features and mechanism, further studies are needed.

## 5. Conclusions

The present results suggest that intermediate-length GGC repeat expansion in *NOTCH2NLC* is associated with ALS. Larger cohorts in different geographic areas are required to further elucidate the characteristics of ALS with intermediate-length GGC, and basic experiments are needed to explore the mechanism of function.

## Figures and Tables

**Table 1:** Demographic characteristics of ALS patients and healthy controls.

	ALS Patients	Healthy Controls
Total	476	210
Male	324 (68.07%)	120 (57.14%)
Female	152 (31.93%)	90 (42.86%)
Age at onset		
Mean ± SD	53.21 ± 11.66	52.58 ± 11.73 ^a^
Site of onset		
Bulbar	87 (18.2%)	-
Spinal	389 (81.7%)	-

Abbreviations: SD: standard deviation. ^a^ Age at enrollment for the control cohort.

**Table 2:** Clinical characteristics of amyotrophic lateral sclerosis patients with intermediate GGC repeat expansion.

Patient.No	Gender	Onset Age (y)	Site of Onset	Diagnosis Delay (mo)	Duration of Disease (mo)	ALS-FRS	*NOTCH2NLC* Repeat Size
1	male	50	Right lower limb	30	35	34	46
2	female	62	bilateral upper limbs	6	30	40	45

Abbreviations: ALS-FRS: ALS Functional Rating Scale.

## Data Availability

The data presented in this study are available upon request from the corresponding author. The data are not publicly available due to data management regulations in our hospital.
